# Interactions of antagonists with subtypes of inositol 1,4,5-trisphosphate (IP_3_) receptor

**DOI:** 10.1111/bph.12685

**Published:** 2014-06-10

**Authors:** Huma Saleem, Stephen C Tovey, Tedeusz F Molinski, Colin W Taylor

**Affiliations:** 1 Department of Pharmacology, University of CambridgeCambridge, UK; 2 Department of Chemistry, University of CaliforniaDavis, CA, USA

**Keywords:** antagonist, 2-APB, caffeine, Ca^2+^ signal, DT40 cell, heparin, inositol 1,4,5-trisphosphate, IP_3_ receptor, structure–activity relationship, Xestospongin

## Abstract

**BACKGROUND AND PURPOSE:**

Inositol 1,4,5-trisphosphate receptors (IP_3_Rs) are intracellular Ca^2+^ channels. Interactions of the commonly used antagonists of IP_3_Rs with IP_3_R subtypes are poorly understood.

**EXPERIMENTAL APPROACH:**

IP_3_-evoked Ca^2+^ release from permeabilized DT40 cells stably expressing single subtypes of mammalian IP_3_R was measured using a luminal Ca^2+^ indicator. The effects of commonly used antagonists on IP_3_-evoked Ca^2+^ release and ^3^H-IP_3_ binding were characterized.

**KEY RESULTS:**

Functional analyses showed that heparin was a competitive antagonist of all IP_3_R subtypes with different affinities for each (IP_3_R3 > IP_3_R1 ≥ IP_3_R2). This sequence did not match the affinities for heparin binding to the isolated N-terminal from each IP_3_R subtype. 2-aminoethoxydiphenyl borate (2-APB) and high concentrations of caffeine selectively inhibited IP_3_R1 without affecting IP_3_ binding. Neither Xestospongin C nor Xestospongin D effectively inhibited IP_3_-evoked Ca^2+^ release via any IP_3_R subtype.

**CONCLUSIONS AND IMPLICATIONS:**

Heparin competes with IP_3_, but its access to the IP_3_-binding core is substantially hindered by additional IP_3_R residues. These interactions may contribute to its modest selectivity for IP_3_R3. Practicable concentrations of caffeine and 2-APB inhibit only IP_3_R1. Xestospongins do not appear to be effective antagonists of IP_3_Rs.

## Introduction

Inositol 1,4,5-trisphosphate receptors (IP_3_R) are intracellular Ca^2+^ channels expressed in the membranes of the endoplasmic reticulum (ER) in most eukaryotic cells (Berridge, 1993; Taylor *et al*., 1999; Foskett *et al*., 2007; nomenclature follows Alexander *et al*., 2013). IP_3_Rs are essential links between the many extracellular signals that stimulate PLC and initiation of cytosolic Ca^2+^ signals triggered by IP_3_-evoked Ca^2+^ release from the ER. Three genes encode closely related IP_3_R subunits in vertebrates, whereas invertebrates have only a single IP_3_R gene (Taylor *et al*., 1999). Each of the three vertebrate IP_3_R subtypes encodes a large polypeptide of about 2700 residues, and they share about 70% amino acid sequence identity (Foskett *et al*., 2007). Within each IP_3_R subunit, IP_3_ binds to a clam-like IP_3_-binding core (IBC; residues 224–604 in IP_3_R1) (Bosanac *et al*., 2002) near the N-terminus. IP_3_ binding to the IBC re-orients its relationship with the associated suppressor domain (residues 1–223). That rearrangement disrupts interactions between adjacent subunits within the tetrameric IP_3_R leading to gating of the Ca^2+^-permeable channel (Seo *et al*., 2012). This central channel of each tetrameric IP_3_R is formed by transmembrane helices and their associated re-entrant loops. These pore-forming structures lie towards the C-terminal of each subunit. How IP_3_-evoked re-arrangement of N-terminal domains of the IP_3_R leads to opening of the pore is not yet resolved, although it is likely to be conserved in all IP_3_R subtypes and broadly similar for the other major family of intracellular Ca^2+^ channels, ryanodine receptors (Seo *et al*., 2012).

Most cells express mixtures of IP_3_R subtypes, although tissues differ in which complements of IP_3_R subunits they express (Taylor *et al*., 1999). Furthermore, the subunits assemble into both homo-tetrameric and hetero-tetrameric structures (Wojcikiewicz and He, 1995). Although all IP_3_Rs are built to a common plan and they are all regulated by IP_3_ and Ca^2+^ (Foskett *et al*., 2007; Seo *et al*., 2012), the subtypes are subject to different modulatory influences (Patterson *et al*., 2004; Higo *et al*., 2005; Foskett *et al*., 2007; Betzenhauser *et al*., 2008; Wagner and Yule, 2012) and they are likely to fulfil different physiological roles (Matsumoto *et al*., 1996; Hattori *et al*., 2004; Futatsugi *et al*., 2005; Tovey *et al*., 2008; Wei *et al*., 2009). It is, however, difficult to disentangle the physiological roles of IP_3_R subtypes in cells that typically express complex mixtures of homo- and hetero-tetrameric IP_3_Rs. There are no ligands of IP_3_Rs that usefully distinguish among IP_3_R subtypes (Saleem *et al*., 2012; 2013) and nor are there effective antagonists that lack serious side effects (Michelangeli *et al*., 1995). Heparin (Ghosh *et al*., 1988), caffeine (Parker and Ivorra, 1991), 2-aminoethoxydiphenyl borate (2-APB) (Maruyama *et al*., 1997) and Xestospongins (Gafni *et al*., 1997) have all been widely used to inhibit IP_3_-evoked Ca^2+^ release, but each has its limitations (see Results). Furthermore, the interactions of these antagonists with IP_3_R subtypes have not been assessed. Peptides derived from myosin light-chain kinase (Nadif Kasri *et al*., 2006; Sun and Taylor, 2008), the N-terminal of IP_3_R1 (Sun *et al*., 2013) or the BH4 domain of bcl-2 (Monaco *et al*., 2012) also inhibit IP_3_-evoked Ca^2+^ release. These peptides are unlikely to provide routes to useful IP_3_R antagonists because they are effective only at high concentrations and they need to be made membrane-permeable. A naturally occurring protein that inhibits IP_3_ binding to IP_3_R, IRBIT (Ando *et al*., 2003), has the same limitations as an experimental tool, and it is effective only when phosphorylated. Many other drugs inhibit IP_3_-evoked Ca^2+^ release, but none of these has found widespread use (see Michelangeli *et al*., 1995; Bultynck *et al*., 2003).

In the present study, we provide the first systematic analysis of the interactions between IP_3_R subtypes and each of the commonly used antagonists. We use DT40 cell lines stably expressing only a single mammalian IP_3_R subtype to define the effects of these antagonists on IP_3_-evoked Ca^2+^ release via each IP_3_R subtype.

## Methods

### Measurement of IP_3_-evoked Ca^2+^ release

We used DT40 cells lacking endogenous IP_3_Rs (Sugawara *et al*., 1997), but stably expressing rat IP_3_R1 (GenBank accession number GQ233032.1; Pantazaka and Taylor, 2011), mouse IP_3_R2 (GU980658.1; Tovey *et al*., 2010) or rat IP_3_R3 (GQ233031.1; Rahman *et al*., 2009). Cells were grown in suspension in RPMI 1640 medium supplemented with 10% FBS, 1% heat-inactivated chicken serum, 2 mM glutamine and 50 μM 2-mercaptoethanol at 37°C in humidified air containing 5% CO_2_. Cells were used or passaged when they reached a density of ∼1.5 × 10^6^ cells mL^−1^.

A low-affinity Ca^2+^ indicator trapped within the ER of permeabilized DT40 cells was used to measure IP_3_-evoked Ca^2+^ release (Tovey *et al*., 2006; Saleem *et al*., 2012). Briefly, the ER was loaded with indicator by incubating cells (∼5 × 10^7^ mL^−1^) in the dark with Mag-fluo-4AM (20 μM) in HEPES-buffered saline (HBS) containing 0.02% (v/v) Pluronic F127 for 1 h at 22°C. HBS had the following composition: 135 mM NaCl, 5.9 mM KCl, 11.6 mM HEPES, 1.5 mM CaCl_2_, 11.5 mM glucose, 1.2 mM MgCl_2_, pH 7.3. After permeabilization of the plasma membrane with saponin (10 μg·mL^−1^, 4 min, 37°C) in Ca^2+^-free cytosol-like medium (CLM), permeabilized cells were washed (650× *g*, 2 min) and resuspended (∼10^7^ mL^−1^) in Mg^2+^-free CLM containing carbonyl cyanide 4-(trifluoromethoxy) phenylhydrazone (FCCP, 10 μM) to inhibit mitochondria, and supplemented with CaCl_2_ to give a final free [Ca^2+^] of 220 nM after addition of 1.5 mM MgATP. Ca^2+^-free CLM had the following composition: 2 mM NaCl, 140 mM KCl, 1 mM EGTA, 20 mM PIPES, 2 mM MgCl_2_, pH 7.0. Permeabilized cells were then distributed into 96-well plates (50 μL, 5 × 10^5^ cells per well), centrifuged (300× *g*, 2 min) and used for experiments at 20°C. Addition of MgATP (1.5 mM) allowed Ca^2+^ uptake by the ER, which was monitored at intervals of ∼1 s using a FlexStation-3 plate reader (MDS Analytical Devices, Berkshire, UK; Tovey *et al*., 2006). After 2 min, when the ER had loaded to steady-state with Ca^2+^, IP_3_ was added with CPA (10 μM) to inhibit further Ca^2+^ uptake. IP_3_-evoked Ca^2+^ release was expressed as a fraction of that released by ionomycin (1 μM; Tovey *et al*., 2006). Similar methods were used to measure IP_3_-evoked Ca^2+^ release from intact or permeabilized HEK cells (Tovey *et al*., 2008). The timings of antagonist additions are described in the figure legends. The affinity of each competitive antagonist (pK_D_) was determined from the intercept on the abscissa of the Schild plot.

Concentration–effect relationships were fitted to Hill equations using Prism (version 5.0, GraphPad, San Diego, CA, USA), from which Hill coefficients (*h*), the fraction of the intracellular Ca^2+^ stores released by a maximally effective concentrations of IP_3_, and pEC_50_ values were calculated.

### Expression of N-terminal fragments of IP_3_ receptors

The plasmids used for bacterial expression of GST-tagged N-terminal fragments (NT, residues 1–604) of rat IP_3_R1, mouse IP_3_R2 and rat IP_3_R3 have been described, and their coding sequences have been confirmed (Khan *et al*., 2013). Plasmids were transformed into BL21-CodonPlus (DE3)-RILP competent cells (Rossi and Taylor, 2011), and grown for 12 h at 37°C in 20 mL of Luria-Bertani (LB) medium containing carbecillin (50 μg·mL^−1^). The volume of medium was then increased to 1 L, and the incubation was continued at 37°C for 3–4 h until the OD_600_ reached 1–1.5. Protein expression was induced by addition of IPTG (0.5 mM) for 20 h at 15°C. Bacteria were harvested (6000× *g*, 5 min), washed twice with cold PBS, and the pellet was suspended (∼10^9^ cells·mL^−1^) in 50 mL of Tris-EDTA medium (TEM: 50 mM Tris, 1 mM EDTA, pH 8.3) supplemented with 10% PopCulture, 1 mM 2-mercaptoethanol and protease inhibitor cocktail (Roche, Burgess Hill, West Sussex, UK; 1 tablet per 50 mL). After lysis by incubation with lysozyme (100 μg·mL^−1^) and RNAse (10 μg·mL^−1^) for 30 min on ice and then sonication (Transsonic T420 water bath sonicator, Camlab, Cambridge, UK; sonicator, 50 Hz, 30 s), the supernatant was recovered (30,000× *g*, 60 min, 4°C). The supernatant was mixed with glutathione Sepharose 4B beads (50:1, v/v, lysate : beads) and incubated with gentle end-over-end rotation (6 rpm) for 45 min at 4°C. The beads were then loaded onto a PD-10 column and washed twice with PBS and twice with PreScission cleavage buffer (GE Healthcare) supplemented with 1 mM DTT. The column was then incubated with 0.5 mL of PreScission cleavage buffer containing 1 mM DTT and 80 units of GST-tagged PreScission protease for 12 h at 4°C using gentle end-over-end rotation. The PreScission protease cuts an engineered cleavage site to release the NT free of its GST tag. The eluted NT (∼15 mg protein mL^−1^) was rapidly frozen and stored at −80°C.

### ^3^H-IP_3_ binding

Equilibrium competition binding assays were performed at 4°C in 500 μL of CLM (final free [Ca^2+^] = 220 nM) containing purified NT (30 μg) or cerebellar membranes (5 mg protein), ^3^H-IP_3_ (1.5 nM) and appropriate concentrations of competing ligand. Reactions were terminated after 5 min by centrifugation (20,000× *g*, 5 min) for membranes, or by centrifugation after addition of poly(ethylene glycol)-8000 [30% (w/v), 500 μL] and γ-globulin (30 μL, 25 mg·mL^−1^) for NT. The pellet was washed (500 μL of 15% PEG or CLM) and solubilized in 200 μL of CLM containing 1% (v/v) Triton-X-100 before liquid scintillation counting. Non-specific binding, whether determined by addition of 10 μM IP_3_ or by extrapolation of competition curves to infinite IP_3_ concentration, was <10% of total binding. Results were fitted to Hill equations using Prism, from which IC_50_ values were calculated. K_D_ (equilibrium dissociation constant) and pK_D_ (–logK_D_) values were calculated from IC_50_ values using the Cheng and Prusoff equation (Cheng and Prusoff, 1973).

### Data analysis

Statistical comparisons used pEC_50_ (or pK_D_) values. For paired comparison of the effect of an antagonist, ΔpEC_50_ values were calculated, where ΔpEC_50_ = 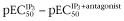. Results are expressed as means ± SEM from *n* independent experiments. Statistical comparisons used paired Student's *t*-test or anova followed by Bonferroni's test, with *P* < 0.05 considered significant.

### Materials

Sources of many reagents were specified in earlier publications (Rossi *et al*., 2010a,b; Saleem *et al*., 2012). IP_3_ was from Enzo Life Sciences (Exeter, UK). ^3^H-IP_3_ (19.3 Ci mmol^−1^) was from PerkinElmer (Buckinghamshire, UK). Heparin (from porcine mucosa, M_r_ 5000) and cyclopiazonic acid (CPA) were from Fisher Scientific (Loughborough, UK). Caffeine, 2-APB, lysozyme, RNAse, γ-globulin and poly(ethylene glycol)-8000 were from Sigma-Aldrich (Dorset, UK). Xestopongins C and D were from Calbiochem (Gibbstown, NJ, USA) or isolated and characterized as previously described (Gafni *et al*., 1997). PopCulture was from Novagen (Darmstadt, Germany). Simply Blue stain was from Invitrogen (Renfrewshire, Scotland). Dioxin-free isopropyl-β-D-thiogalactoside (IPTG), and Luria–Bertani agar and broth were from Formedium (Norfolk, UK). Glutathione Sepharose 4B beads and GST-tagged PreScission protease were from GE Healthcare (Buckinghamshire, UK). Carbecillin was from Melford Laboratories (Suffolk, UK). BL21-CodonPlus (DE3)-RILP competent bacteria were from Agilent Technology (Berkshire, UK).

## Results

### Heparin is a competitive antagonist with different affinities for IP_3_ receptor subtypes

**Figure 1:**
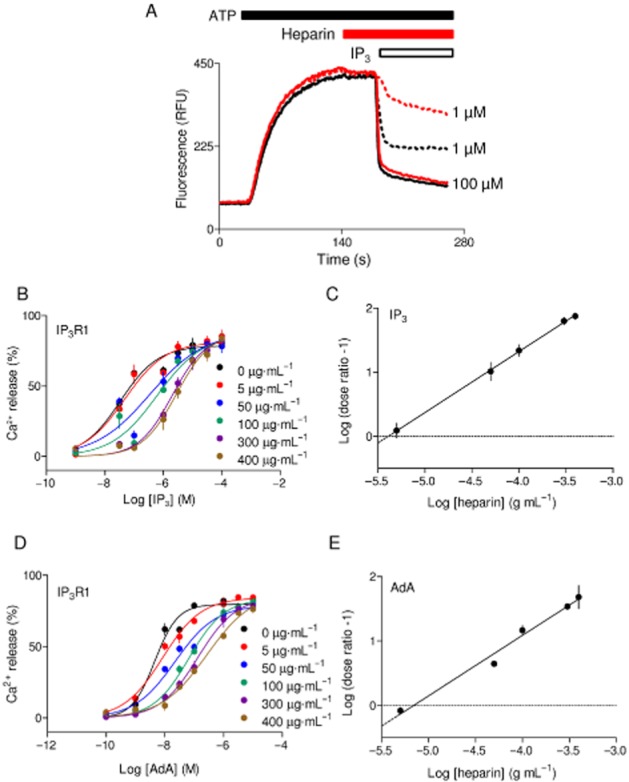
Heparin competitively inhibits IP_3_-evoked Ca^2+^ release via type 1 IP_3_ receptors. (A) Typical traces from a population of permeabilized DT40-IP_3_R1 cells showing the fluorescence (RFU, relative fluorescence units) recorded from a luminal Ca^2+^ indicator after addition of MgATP (1.5 mM), heparin (400 μg·mL^−1^, red lines; or CLM alone, black lines) and then IP_3_ (1 or 100 μM). The traces show average responses from two wells in a single plate. (B) Experiments similar to those in A show concentration-dependent effects of IP_3_ on Ca^2+^ release in the presence of the indicated concentrations of heparin. (C) Schild analysis of the results shown in B. (D, E) Similar analyses of the effects of heparin on AdA-evoked Ca^2+^ release via IP_3_R1. Results (B–E) are means ± SEM from three experiments.

Heparin is a competitive antagonist of IP_3_-evoked Ca^2+^ release (Ghosh *et al*., 1988), but it is membrane-impermeable and it has many additional effects. These include uncoupling of receptors from G-proteins (Willuweit and Aktories, 1988; Dasso and Taylor, 1991), stimulation of ryanodine receptors (Ehrlich *et al*., 1994) and inhibition of IP_3_ 3-kinase (Guillemette *et al*., 1989). To assess the effects of heparin on each IP_3_R subtype, permeabilized DT40 cells expressing each of the three IP_3_R subtypes were incubated with heparin for 35 s. The effect of IP_3_ on Ca^2+^ release from the intracellular stores was then assessed (Figure 1A). In permeabilized DT40-IP_3_R1 cells, heparin caused parallel rightward shifts of the concentration–response relationship for IP_3_-evoked Ca^2+^ release (Figure 1B). Schild plots, which had slopes of 0.95 ± 0.02 (mean ± SEM, *n* = 3), established that the equilibrium dissociation constant (K_D_) for heparin was 4.1 μg·mL^−1^ (pK_D_ = 5.39 ± 0.00) (Figure 1C). Similar results were obtained when adenophostin A (AdA), a high-affinity agonist of IP_3_Rs (Rossi *et al*., 2010b; Saleem *et al*., 2013), was used to stimulate Ca^2+^ release. The Schild plots had slopes of 0.94 ± 0.03 (*n* = 3) and the K_D_ for heparin was 6.9 μg·mL^−1^ (pK_D_ = 5.16 ± 0.05) (Figure 1D and E; Table 1).

**Table 1:** Effects of heparin on IP_3_-evoked Ca^2+^ release and IP_3_ binding

		Functional analysis		aBinding	pEC_50_(IP_3_)-pK_D_(heparin)
		IP_3_ or AdA	heparin	heparin	
		pEC_50_	pK_D_	pK_D_	
IP_3_R1	IP_3_	7.47 ± 0.02	5.39 ± 0.00	4.66	2.08 ± 0.02
IP_3_R1	AdA	8.35 ± 0.03	5.16 ± 0.05	–	–
IP_3_R2	IP_3_	6.82 ± 0.04	4.66 ± 0.07	4.62	2.16 ± 0.09*
IP_3_R3	IP_3_	6.66 ± 0.07	5.55 ± 0.09	5.34	1.11 ± 0.08*
IP_3_R3	AdA	7.71 ± 0.01	5.68 ± 0.04	–	–

**Figure 2:**
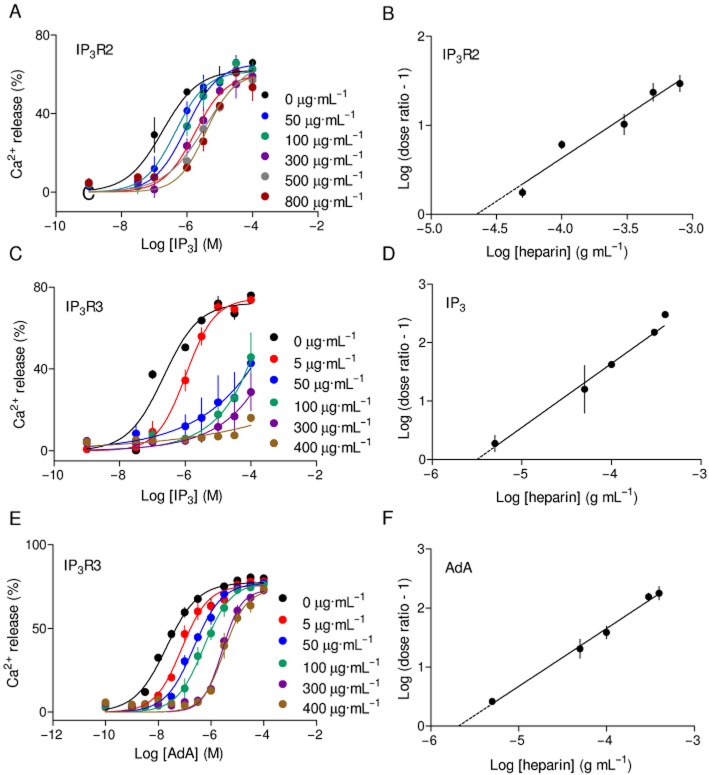
Heparin is a competitive antagonist with different affinities for types 2 and 3 IP_3_ receptors. (A) Concentration-dependent release of Ca^2+^ by IP_3_ from the intracellular stores of DT40-IP_3_R2 cells in the presence of the indicated concentrations of heparin added 35 s before IP_3_. (B) Schild plot of the results. (C–F) Similar analyses of DT40-IP_3_R3 cells stimulated with IP_3_ (C, D) or AdA (E, F). For D, where maximal attainable concentrations of IP_3_ were insufficient to evoke maximal responses in the presence of the highest concentrations of heparin, the Schild plot shows dose ratios calculated from IP_3_ concentrations that evoked 40% Ca^2+^ release. Results (A–F) are mean ± SEM from three experiments.

From experiments similar to those shown in Figures 1 and 2, AdA or IP_3_-evoked Ca^2+^ release and their sensitivity to heparin were used to determine pEC_50_ (as M) and pK_D_ (as g mL^−1^) for DT40 cells expressing IP_3_R1, IP_3_R2 or IP_3_R3. Results are means ± SEM from three independent experiments (six for IP_3_R3).

a The affinities for heparin determined from equilibrium-competition binding with ^3^H-IP_3_ to Sf9 membranes expressing IP_3_R1-3 are reproduced from (Nerou *et al*., 2001). The batch of heparin used for those binding studies was different from that used for the work reported here. The final column (derived from the results shown in Figures 1B,C and 2A–D) shows paired comparisons of pEC_50_(IP_3_) – pK_D_(heparin) as a means of reporting the relative effectiveness with which heparin might be expected to block IP_3_-evoked Ca^2+^ release via different IP_3_R subtypes. The results suggest that IP_3_R3 is likely to be substantially more susceptible to inhibition than IP_3_R1 or IP_3_R2.

* Denotes a value significantly different from IP_3_R1 in the final column (*P* < 0.05).

A similar analysis of the effects of heparin on IP_3_-evoked Ca^2+^ release from permeabilized DT40-IP_3_R2 cells was also consistent with competitive antagonism. The slope of the Schild plots was 0.97 ± 0.06 (*n* = 3) and the K_D_ for heparin was 22 μg·mL^−1^ (pK_D_ = 4.66 ± 0.07) ( Figure 2A and B). IP_3_R3 are less sensitive to IP_3_ than the other subtypes (Iwai *et al*., 2007; Saleem *et al*., 2013) (Table 1). This made it difficult to add IP_3_ at concentrations sufficient to achieve maximal Ca^2+^ release in the presence of heparin concentrations greater than 5 μg·mL^–1^ (Figure 2C). Assuming the maximal response to IP_3_ was unaffected by heparin, we used the concentrations of IP_3_ that evoked release of 40% of the intracellular stores to construct Schild plots for IP_3_R3. The results were consistent with competitive antagonism. The slope of the Schild plots was 1.14 ± 0.41 (*n* = 3) and the K_D_ for heparin was 2.8 μg·mL^−1^ (pK_D_ = 5.55 ± 0.09) (Figure 2D and Table 1). AdA has ∼10-fold higher affinity than IP_3_ for all three IP_3_R subtypes (Table 1) (Rossi *et al*., 2010a; Saleem *et al*., 2013), and we have shown that the affinity of heparin for IP_3_R1 is similar whether IP_3_ or AdA is used to evoke Ca^2+^ release (Figure 1B–E). To obtain an independent measure of the affinity of IP_3_R3 for heparin, free of the problems associated with using IP_3_, we therefore repeated the Schild analysis using AdA to stimulate Ca^2+^ release. These conditions provided complete concentration–effect relationships for AdA at a wider range of heparin concentrations (Figure 2E). The Schild plots had a slope of 0.98 ± 0.04 (*n* = 6) and the K_D_ for heparin was 2.1 μg·mL^−1^ (pK_D_ = 5.68 ± 0.04) (Figure 2F and Table 1). The affinity of heparin for IP_3_R3 was therefore similar whether measured using IP_3_ or AdA to evoke Ca^2+^ release.

These functional analyses establish that heparin is a competitive antagonist of IP_3_ at all three IP_3_R subtypes, but with different affinities for each (IP_3_R3 > IP_3_R1 ≥ IP_3_R2) (Table 1). The results are consistent with an analysis of IP_3_ binding to mammalian IP_3_R expressed in Sf9 cells (Nerou *et al*., 2001), where the pK_D_ values and rank order of heparin affinity (IP_3_R3 > IP_3_R1 ∼ IP_3_R2) were similar to those from the present functional analyses (Table 1).

### Heparin binding is not solely determined by its interactions with the IP_3_-binding site

**Figure 3:**
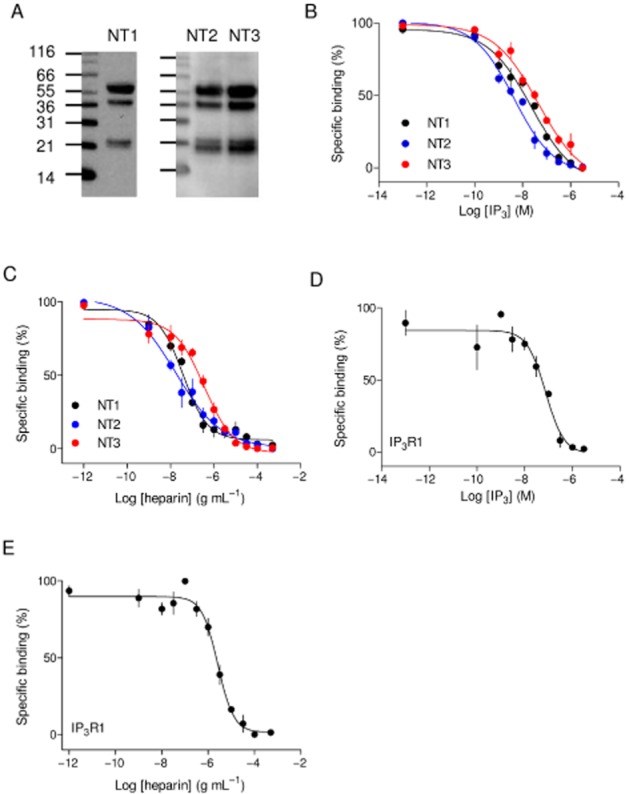
Heparin binding is not solely determined by its interactions with the IP_3_-binding core. (A) Immunoblots of purified NT1-3 (∼15 μL protein per lane) using an antiserum that recognizes a conserved sequence within all three IP_3_R subtypes (residues 62–75 in rat IP_3_R1). The positions of M_r_ markers (kDa) are shown alongside each blot. (B, C) Equilibrium-competition binding of IP_3_ (B) and heparin (C) to purified NT1-3 in CLM. (D, E) Similar analyses of binding to cerebellar membranes (IP_3_R1). Results (B–E) are means ± SEM from three to six experiments.

Activation of IP_3_Rs is initiated by binding of IP_3_ to the IP_3_-binding core (IBC, residues 224-604 of IP_3_R1) within the N-terminal region of each IP_3_R subunit (see Introduction) (Seo *et al*., 2012). The only contacts between IP_3_ and the IP_3_R are mediated by residues within the IBC (Bosanac *et al*., 2002), but interaction of the N-terminal suppressor domain (residues 1-223) with the IBC reduces its affinity for IP_3_. Hence, the IBCs from different IP_3_R subtypes bind IP_3_ with similar affinity, whereas the larger N-terminal regions (NT, residues 1-604) have lower affinities that differ between subtypes. The NTs bind IP_3_ with two- to threefold greater affinities than those of full-length IP_3_Rs, but the NTs and full-length IP_3_Rs have the same rank order of affinities for IP_3_ (NT2 > NT1 > NT3) (Iwai *et al*., 2007; Rossi *et al*., 2009). The results shown in Figure 3A and B, which show IP_3_ binding to bacterially expressed NTs from each of the three IP_3_R subtypes (NT1-3), confirm previous results. Surprisingly, however, equilibrium-competition binding of heparin to NTs in medium that matches that used to measure IP_3_-evoked Ca^2+^ release was not consistent with the results obtained from functional analyses (Figure 3C). The affinity of the NT for heparin was up to 2000-fold greater than that measured in functional analyses, and the rank order of affinity for heparin was different for NTs (NT2 > NT1 > > NT3) and full-length IP_3_Rs (IP_3_R3 > IP_3_R1 ≥ IP_3_R2) (Nerou *et al*., 2001; Tables 1 and 2).

**Table 2:** Heparin and IP_3_ binding to N-terminal fragments of IP_3_ receptor subtypes

	NT1	NT2	NT3	IP_3_R1
IP_3_	7.76 ± 0.07	8.67 ± 0.15	7.39 ± 0.08	7.13 ± 0.08
Heparin	7.42 ± 0.09	7.95 ± 0.32	6.59 ± 0.09*	5.61 ± 0.13

Equilibrium-competition binding with ^3^H-IP_3_ was used to measure pK_D_ values for IP_3_ (as M) and heparin (as g mL^−1^) binding to purified NT1-3 and cerebellar membranes (IP_3_R1). Results are means ± SEM from three to six experiments.

* Denotes a significant difference from NT1 (*P* < 0.05) for 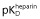.

IP_3_R1 is the major (>99%) subtype in cerebellar membranes (Wojcikiewicz, 1995). Equilibrium-competition binding of heparin to cerebellar membranes in CLM established that the affinity of IP_3_R1 for heparin (pK_D_ = 5.61 ± 0.13, *n* = 3) was similar to that derived from Schild analysis of DT40-IP_3_R1 cells (pK_D_ = 5.39 ± 0.00, *n* = 3) and similar to that reported for heparin binding to IP_3_R1 heterologously expressed in Sf9 cells (Nerou *et al*., 2001), but very different to the heparin affinity of NT1 (pK_D_ = 7.42 ± 0.09, *n* = 3) (Tables 1 and 2). These results demonstrate that the IBC is not the only determinant of competitive heparin binding to IP_3_Rs and suggest either that access of heparin to the IBC is influenced by additional interactions or that heparin binding to an additional site affects IP_3_R gating.

### 2-APB selectively inhibits Ca^2+^ release via type 1 IP_3_ receptors without affecting IP_3_ binding

**Figure 4:**
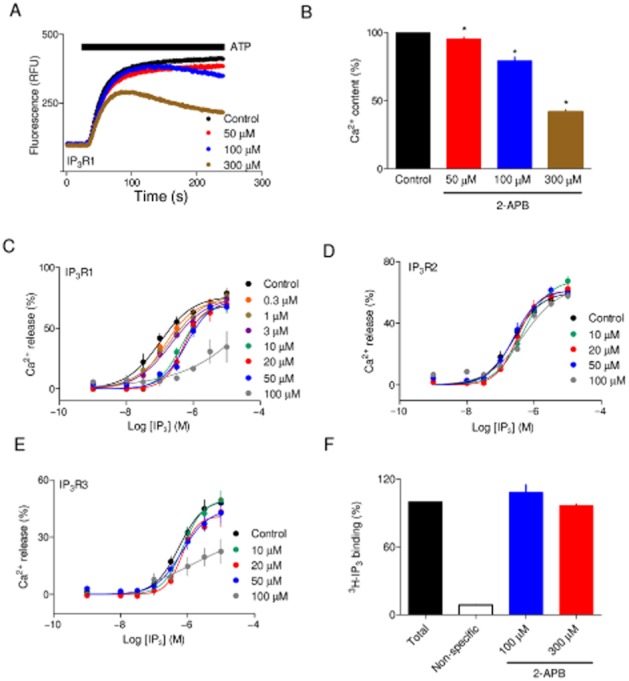
2-APB selectively inhibits Ca^2+^ release via type 1 IP_3_ receptors. (A) Ca^2+^ uptake into the intracellular stores of permeabilized DT40-IP_3_R1 cells is shown after addition of ATP in the presence of the indicated concentrations of 2-APB. Each trace is the average from two wells in a single plate. (B) Summary results show effects of 2-APB on Ca^2+^ contents measured 180 s after addition of ATP. (C–E) Concentration-dependent effects of IP_3_ on Ca^2+^ release from permeabilized DT40-IP_3_R1-3 cells alone or with the indicated concentrations of 2-APB added 35 s before IP_3_. (F) Binding of ^3^H-IP_3_ (1.5 nM) to cerebellar membranes (IP_3_R1), with 3 μM IP_3_ (non-specific) or with 2-APB. Results (B–F) are means ± SEM from three to nine experiments. *P < 0.05, significantly different from control.

2-APB is membrane-permeant and is often used to inhibit IP_3_-evoked Ca^2+^ release (Maruyama *et al*., 1997; Missiaen *et al*., 2001; Bilmen *et al*., 2002), but it has many additional effects. These include modulation of store-operated Ca^2+^ entry (Goto *et al*., 2010) and inhibition of the sarcoplasmic/endoplasmic reticulum Ca^2+^-ATPase (SERCA) that mediates Ca^2+^ sequestration by the ER (Missiaen *et al*., 2001; Bilmen *et al*., 2002; Bultynck *et al*., 2003). In permeabilized DT40-IP_3_R1 cells, 50 μM 2-APB had no effect on Ca^2+^ uptake by the ER, although higher concentrations reduced the steady-state Ca^2+^ content (Figure 4A and B). This is consistent with high concentrations of 2-APB causing inhibition of SERCA.

In permeabilized DT40-IP_3_R1 cells, 2-APB caused a concentration-dependent inhibition of IP_3_-evoked Ca^2+^ release (Figure 4C). With 50 μM 2-APB, the highest concentration that avoids inhibition of Ca^2+^ uptake, there was an approximately sevenfold decrease in IP_3_ sensitivity (ΔpEC_50_ = 0.84 ± 0.12) with no effect on the maximal response to IP_3_ (Figure 4C). The same concentration of 2-APB (50 μM) had no significant effect on IP_3_-evoked Ca^2+^ release from permeabilized DT40-IP_3_R2 or DT40-IP_3_R3 cells (Figure 4D and E). When the 2-APB concentration was increased to 100 μM, which caused some inhibition of Ca^2+^ uptake (Figure 4A and B), there was some inhibition of IP_3_R3, but no effect on IP_3_-evoked Ca^2+^ release via IP_3_R2 (Figure 4D and E; Table 3).

**Table 3:** Selective inhibition of IP_3_ receptor subtypes by common antagonists

	IP_3_R1		IP_3_R2		IP_3_R3	
	ΔpEC_50_ (M)	ΔMax (%)	ΔpEC_50_ (M)	ΔMax (%)	ΔpEC_50_ (M)	ΔMax (%)
Heparin, 400 μg·mL^−1^	1.88 ± 0.05*	−7 ± 2	ND	–	2.34 ± 0.07*	−3 ± 3
Heparin, 800 μg·mL^−1^	ND	–	1.49 ± 0.09*	−4 ± 2	ND	–
Caffeine, 70 mM	0.61 ± 0.07*	12 ± 4	−0.2 ± 0.07	−1 ± 0	−0.07 ± 0.08	0 ± 5
2-APB, 50 μM	0.84 ± 0.12*	0 ± 4	−0.05 ± 0.10	0 ± 4	0.02 ± 0.09	8 ± 4
Xestospongin C, 20 μM	0.21 ± 0.10*	6 ± 2*	−0.06 ± 0.04	1 ± 1	0.12 ± 0.03*	1 ± 2
Xestospongin D, 20 μM	0.26 ± 0.09*	18 ± 2*	−0.15 ± 0.05	8 ± 3*	0.21 ± 0.10*	2 ± 2

Summary of the functional analyses of antagonists on IP_3_-evoked Ca^2+^ release from permeabilized DT40-IP_3_R1-3 cells. The pEC_50_ values for IP_3_ and the maximal Ca^2+^ release are each expressed relative to the response evoked in paired controls without antagonist (Δ = control – response with antagonist). A positive Δ value demonstrates an inhibition of IP_3_-evoked Ca^2+^ release by the antagonist. The results with Xestospongins C and D are pooled from experiments that included pre-incubation periods of 7 and 12 min (see Supporting Information Table S1). Results are means ± SEM from three to nine experiments.

* Denotes a value significantly greater than 0 (*P* < 0.025, one-tailed test).

ND, not determined.

Binding of ^3^H-IP_3_ to IP_3_R1 of cerebellar membranes in CLM was unaffected by 2-APB (Figure 4F) consistent with published results (Maruyama *et al*., 1997; Bilmen *et al*., 2002). This demonstrates that inhibition of IP_3_R1 by 2-APB is neither due to competition with IP_3_ nor to allosteric inhibition of IP_3_ binding.

### Caffeine is a low-affinity antagonist of type 1 IP_3_ receptors

**Figure 5:**
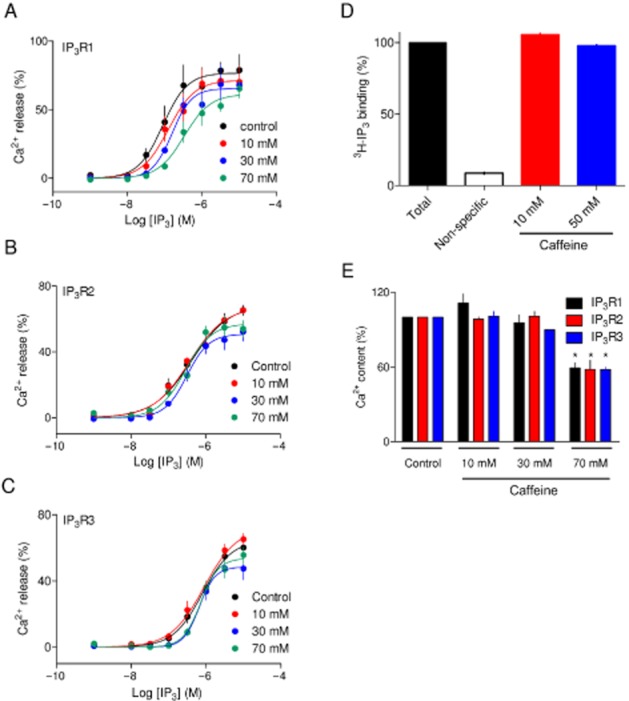
Caffeine is a low-affinity antagonist of type 1 IP_3_R receptors. (A–C) Concentration-dependent effects of IP_3_ on Ca^2+^ release from permeabilized DT40-IP_3_R1-3 cells in the presence of the indicated concentrations of caffeine added 4 min before IP_3_. (D) Binding of ^3^H-IP_3_ (1.5 nM) to cerebellar membranes alone (total), with 3 μM IP_3_ (non-specific) or caffeine. (E) Effect of caffeine added 2 min before ATP on the steady-state Ca^2+^ content of the intracellular stores (percentage of matched control cells) measured 90 s after addition of ATP to DT40-IP_3_R1-3 cells. Results (A–E) are means ± SEM from three experiments. *P < 0.05 significantly different from control.

Caffeine is another membrane-permeant antagonist of IP_3_-evoked Ca^2+^ release (Parker and Ivorra, 1991; Brown *et al*., 1992; Bultynck *et al*., 2003; Laude *et al*., 2005), but it is effective only at high (mM) concentrations and it has many additional effects (Michelangeli *et al*., 1995; Taylor and Tovey, 2010). These include stimulation of ryanodine receptors, inhibition of cyclic nucleotide phosphodiesterases, competitive antagonism of adenosine receptors, and effects on the fluorescence of some Ca^2+^ indicators (Brown *et al*., 1992; Ehrlich *et al*., 1994; Michelangeli *et al*., 1995; McKemy *et al*., 2000; Taylor and Tovey, 2010). High concentrations of caffeine (10–70 mM) inhibited Ca^2+^ release via IP_3_R1 (Figure 5A) without affecting ^3^H-IP_3_ binding to cerebellar membranes (Figure 5D). The latter is consistent with published work (Brown *et al*., 1992). The maximal attainable concentration of caffeine (70 mM) caused an approximately fourfold decrease in IP_3_ sensitivity (ΔpEC_50_ = 0.61 ± 0.07) (Figure 5A). Caffeine had no significant effect on IP_3_-evoked Ca^2+^ release via IP_3_R2 or IP_3_R3 (Figure 5B and C; Table 3). At the highest concentration used (70 mM), caffeine significantly reduced the Ca^2+^ content of the intracellular stores, but this inhibition was similar for DT40 cells expressing each of the IP_3_R subtypes (Figure 5E). Inhibition of Ca^2+^ sequestration by the ER is unlikely, therefore, to account for the selective inhibition of IP_3_-evoked Ca^2+^ release via IP_3_R1 (Table 3). These results demonstrate that a high concentration of caffeine modestly, but selectively, inhibits IP_3_-evoked Ca^2+^ release via IP_3_R1 without affecting IP_3_ binding.

### Xestospongins do not effectively inhibit IP_3_-evoked Ca^2+^ release

Xestospongin C is membrane-permeant and was reported to inhibit IP_3_-evoked Ca^2+^ release from cerebellar microsomes (IC_50_ = 358 nM) without affecting IP_3_ binding (Gafni *et al*., 1997). Xestospongin D is less potent. Higher concentrations of Xestospongin C (10–20 μM) were required to inhibit IP_3_-evoked Ca^2+^ release in intact cells. We assessed the effects of Xestospongins C and D from different suppliers (see Materials) on Ca^2+^ release mediated by each of the three IP_3_R subtypes.

**Figure 6:**
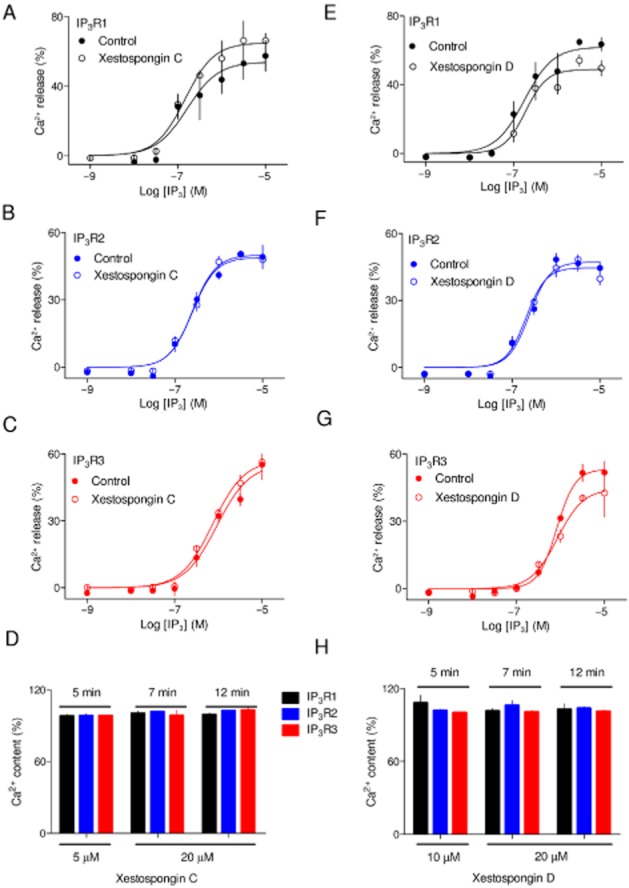
Xestospongins do not effectively inhibit IP_3_ receptors. (A–C) IP_3_-evoked Ca^2+^ release from permeabilized DT40-IP_3_R1-3 cells is shown with or without 5 μM Xestospongin C (from Gafni *et al*., 1997) added 5 min before IP_3_. (D) Effects of Xestospongin C (5–20 μM) added 5–12 min before ATP on the Ca^2+^ content of the intracellular stores (percentages of matched controls without Xestospongin). (E–H) Similar analyses using Xestospongin D (10 μM added 5 min before IP_3_). Results (A–H) are means ± SEM from three experiments.

Pre-incubation of permeabilized DT40 cells with Xestospongin C (5–20 μM from either source) for 5–12 min before addition of IP_3_ had no significant effect on IP_3_-evoked Ca^2+^ release mediated by any of the three IP_3_R subtypes (Supporting Information Table S1). Figure 6A–C show IP_3_-evoked Ca^2+^ release after a 5 min pre-incubation with 5 μM purified Xestospongin C (Gafni *et al*., 1997). It had no significant effect on either the response to IP_3_ (Figure 6A–C) or the Ca^2+^ content of the stores (Figure 6D). Pooling all experiments with the highest concentration of Xestospongin C (20 μM, *n* = 6) revealed a statistically significant (*P* < 0.025, one-tailed test), but very small, inhibition of the maximal response from IP_3_R1, and an even smaller increase in pEC_50_ for IP_3_R1 and IP_3_R3 (Table 3 and Supporting Information Table S1).

Similar treatments with Xestospongin D (10–20 μM from either source) for 5–12 min caused a modest, but statistically significant (*P* < 0.025, one-tailed test), inhibition of IP_3_-evoked Ca^2+^ release via IP_3_R1 (Supporting Information Table S1). Figure 6E–H show that a 5 min pre-incubation with 10 μM purified Xestospongin D (Gafni *et al*., 1997) had no effect on the Ca^2+^ content of the intracellular stores, but modestly inhibited IP_3_-evoked Ca^2+^ release via IP_3_R1 (*P* < 0.025, one-tailed test, Figure 6E). Pooling results with the highest concentration of Xestospongin D (20 μM, *n* = 6) revealed a statistically significant (*P* < 0.025, one-tailed test), but very small, inhibition of the maximal response from IP_3_R1 and IP_3_R2, and a tiny increase in the pEC_50_ for IP_3_R1 and IP_3_R3 (Table 3 and Supporting Information Table S1). These small inhibitory effects of Xestospongins C and D are not sufficient to be useful, and nor are they sufficient to reliably assess whether there is any subtype-selective interaction of Xestospongins with IP_3_Rs.

**Figure 7:**
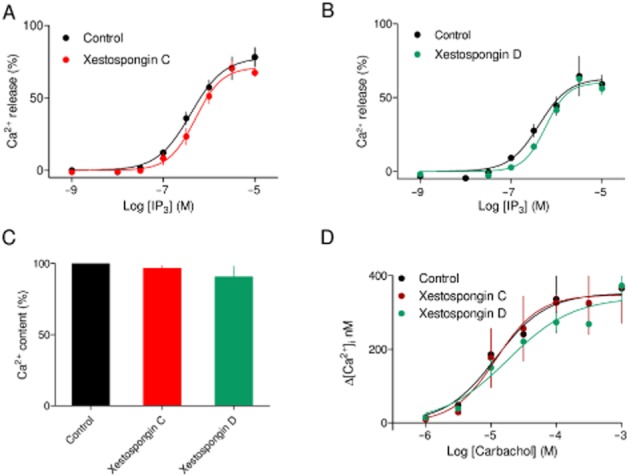
Xestospongins do not inhibit IP_3_-evoked Ca^2+^ signals in HEK cells. (A–C) Permeabilized HEK cells were incubated with Xestospongin C (5 μM) or Xestospongin D (10 μM) for 5 min before addition of IP_3_. Both Xestospongins were prepared as described (Gafni *et al*., 1997). Results show IP_3_-evoked Ca^2+^ release (A, B) or the steady-state Ca^2+^ content of the intracellular stores (C, as a percentage of matched controls without Xestospongin). (D) Concentration-dependent effects of carbachol on the increase in intracellular free Ca^2+^ concentration [Ca^2+^]_i_ of intact fluo-4-loaded HEK cells after treatment with Xestospongins C or D (10 μM for 30 min). pEC_50_ (M) values for the carbachol-evoked Ca^2+^ signals were 4.99 ± 0.13, 4.92 ± 0.23 and 4.70 ± 0.11 for control cells and cells treated with Xestospongins C and D respectively. Results (A–D) are means ± SEM. from three experiments.

We also assessed the effects of Xestospongins on IP_3_-evoked Ca^2+^ release from intact and permeabilized HEK cells. IP_3_ caused a concentration-dependent release of Ca^2+^ from the intracellular stores of permeabilized HEK cells (Figure 7A and B). Pre-incubation of the permeabilized cells for 5 min with Xestospongin C (5 μM) or Xestospongin D (10 μM) had no effect on the Ca^2+^ content of the intracellular stores (Figure 7C) or the Ca^2+^ release evoked by IP_3_ (Figure 7A and B). Carbachol, via endogenous M_3_ muscarinic receptors of HEK cells, stimulates PLC and thereby IP_3_-evoked Ca^2+^ release. Preincubation of HEK cells with Xestospongin C or D (10 μM) for 30 min had no significant effect on the Ca^2+^ signals evoked by any concentration of carbachol (Figure 7D). This conflicts with published results from similar experiments, where Xestospongin C (10 μM for 30 min) caused substantial, though incomplete, inhibition of carbachol-evoked Ca^2+^ signals (Kurian *et al*., 2009). It is, however noteworthy, in light of evidence that Xestospongins have been reported to inhibit Ca^2+^ uptake into the ER (Castonguay and Robitaille, 2002; Solovyova *et al*., 2002), that in the experiments from Kurian *et al*. HEK cells were incubated with Xestospongin for 30 min in Ca^2+^-free medium, while in our experiments extracellular free Ca^2+^ was removed immediately before stimulation with carbachol. The discrepant results may, therefore, reflect an increased loss of Ca^2+^ from intracellular stores during prolonged exposure to Xestospongin in Ca^2+^-free medium.

## Discussion

Acute analyses of IP_3_-evoked Ca^2+^ signalling are handicapped by lack of effective and selective antagonists (Michelangeli *et al*., 1995; Bultynck *et al*., 2003). Furthermore, the subtype-selectivity and in many cases the mechanism of action of the antagonists that are routinely used are not known. We have addressed these issues by examining the functional effects of the most widely used antagonists of IP_3_R in cells expressing only a single IP_3_R subtype.

Heparin is a competitive antagonist of IP_3_ at cerebellar IP_3_Rs (Ghosh *et al*., 1988), most likely because as a polyanion it may partially mimic the phosphate groups of IP_3_. That is consistent with evidence that other polyanions, like decavanadate, ATP and dextran sulphate, can also competitively inhibit IP_3_Rs (Bultynck *et al*., 2003). Our functional analyses establish that heparin is a competitive antagonist of all three IP_3_R subtypes, but with modestly different affinities for each (IP_3_R3 > IP_3_R1 ≥ IP_3_R2) (Figures 1 and 2; Table 1). The affinities of IP_3_R subtypes for heparin derived from functional analyses were similar to those determined from equilibrium-competition binding to native IP_3_R1 (Figure 3E) or to heterologously expressed IP_3_R subtypes (Table 1). However, heparin bound to N-terminal fragments (NT) of IP_3_Rs that include the IBC with an affinity that was up to 2000-fold greater than its affinity for the corresponding full-length IP_3_R (Tables 1 and 2). Furthermore, the rank order of heparin affinity for IP_3_R1-3 and NT1-3 was different. We conclude that heparin inhibits IP_3_-evoked Ca^2+^ release by competing with IP_3_, but its access to the IBC is substantially impaired in full-length IP_3_Rs within native membranes. Phospholipids may contribute to the substantially lesser affinity of heparin for IP_3_R in native membranes by electrostatically repelling the approach of polyanionic heparin to the membrane-bound IBC. In addition, we suggest that charged residues on the IP_3_R surface may differentially influence heparin access to the IBC of each IP_3_R subtype and thereby contribute to the modestly different affinities of heparin for IP_3_R subtypes (Table 1). Our observations have more general significance for analyses of competitive antagonism. We have demonstrated that properties of either the receptor or its environment that are remote from the ligand-binding site may significantly affect the apparent affinity of a receptor for a competitive antagonist.

Because heparin is a competitive antagonist of IP_3_ (Figures 1 and 2), its experimental utility will depend on its affinity relative to IP_3_ for each IP_3_R subtype. Table 1 addresses this issue by comparing measured affinities for heparin with EC_50_ values for IP_3_ as an estimate of the relative affinity of each IP_3_R subtype for IP_3_. The analysis indicates that within native cells, responses of IP_3_R3 to IP_3_ are likely to be more susceptible to inhibition by heparin than the responses mediated by other IP_3_R subtypes.

Both 2-APB and caffeine selectively inhibited IP_3_-evoked Ca^2+^ release via IP_3_R1, without affecting IP_3_ binding (Figures 4 and 5; Table 3). Higher concentrations of 2-APB caused some inhibition of IP_3_R3, but this was accompanied by inhibition of ER Ca^2+^ uptake (Figure 4). The highest concentration of caffeine used (70 mM) also inhibited Ca^2+^ sequestration by the ER, but without significantly affecting the sensitivity to IP_3_ of IP_3_R2 or IP_3_R3, or the fraction of the remaining Ca^2+^ stores released via them by a maximally effective concentration of IP_3_ (Figure 5). Previous analyses of cells expressing different mixtures of native IP_3_R subtypes have also suggested that IP_3_R2 may be resistant to inhibition by 2-APB (Gregory *et al*., 2001; Hauser *et al*., 2001; Kukkonen *et al*., 2001; Bootman *et al*., 2002; Soulsby and Wojcikiewicz, 2002) and caffeine (Kang *et al*., 2010). The mechanism of action of 2-APB is unresolved, but for IP_3_R1 caffeine appears to compete with ATP for the site through which ATP potentiates IP_3_-evoked Ca^2+^ release (Missiaen *et al*., 1994; Maes *et al*., 2001). This mechanism appears not to explain the actions of 2-APB (Missiaen *et al*., 2001). ATP potentiates IP_3_-evoked Ca^2+^ release via all three IP_3_R subtypes (Smith *et al*., 1985; Mak *et al*., 1999; Maes *et al*., 2001; Tu *et al*., 2005; Betzenhauser *et al*., 2008), but the mechanisms and ATP-binding sites differ (Betzenhauser *et al*., 2008; 2009; Betzenhauser and Yule, 2010). Work from Yule and his colleagues suggests that IP_3_R2 is most sensitive to ATP and for it, but not other IP_3_R subtypes, an ATPB site within each IP_3_R subunit mediates the potentiating effect of ATP (Betzenhauser and Yule, 2010). It is, therefore, tempting to speculate that the different sensitivities of IP_3_R subtypes to inhibition by caffeine (Figure 5) may be related to their different modes of regulation by ATP.

Xestospongins were initially shown to inhibit IP_3_-evoked Ca^2+^ release selectively (Gafni *et al*., 1997), and numerous subsequent analyses of their effects on intact cells are consistent with inhibition of IP_3_Rs (e.g. Bishara *et al*., 2002; Duncan *et al*., 2007; Oka *et al*., 2002; Ozaki *et al*., 2002; Rosado and Sage, 2000; Schafer *et al*., 2001; Yuan *et al*., 2005), but few of these later analyses directly addressed the effects of Xestospongins on IP_3_Rs (e.g. Oka *et al*., 2002; Ozaki *et al*., 2002). The latter is important because Xestospongins have additional effects that include inhibition of SERCA (De Smet *et al*., 1999; Castonguay and Robitaille, 2002; Solovyova *et al*., 2002), store-operated Ca^2+^ entry (Bishara *et al*., 2002), L-type Ca^2+^ channels and Ca^2+^-activated K^+^ channels (Ozaki *et al*., 2002), and modulation of ryanodine receptors (Ta *et al*., 2006). The potencies of Xestospongins also differ between studies and some reports challenge whether they effectively inhibit IP_3_Rs (Solovyova *et al*., 2002; Duncan *et al*., 2007; Govindan and Taylor, 2012). We used two sources of Xestospongins C and D, a range of concentrations and incubation periods, two different cell types (see also Govindan and Taylor, 2012), and both intact and permeabilized cells. Although the Xestospongins caused some inhibition of IP_3_-evoked Ca^2+^ release, none of our analyses succeeded in demonstrating that attainable (≤20 μM) concentrations of Xestospongins substantially inhibited any IP_3_R subtype (Figures 6 and 7; Table 3; Supporting Information Table S1).

We conclude that none of the commonly used antagonists of IP_3_Rs is free of pitfalls. Heparin is perhaps the most reliable, it is competitive with IP_3_, but it is membrane-impermeant, and its binding to the IBC of IP_3_Rs is influenced by more distant residues that cause it to bind with different affinity to each IP_3_R subtype (Figures 3). Caffeine and 2-APB are membrane-permeant, they do not compete with IP_3_, but neither achieves effective inhibition of IP_3_Rs without affecting other Ca^2+^-regulating proteins, and both show selectivity for IP_3_R1 (Figures 4 and 5). Xestospongins are membrane-permeant and reported to inhibit IP_3_-evoked Ca^2+^ release without affecting IP_3_ binding (Gafni *et al*., 1997), but in our hands they do not inhibit any IP_3_R subtype (Figures 6 and 7).

## Abbreviations

2-APB: 2-aminoethoxydiphenyl borate
AdA: adenophostin A
CLM: cytosol-like medium
CPA: cyclopiazonic acid
ER: endoplasmic reticulum
HBS: HEPES-buffered saline
IBC: IP_3_-binding core (residues 224-604)
IP_3_: inositol 1,4,5-trisphosphate
IPTG: isobutyl-β-D-thiogalactoside
NT1-3: residues 1-604 of IP_3_R1-3
SERCA: sarcoplasmic/endoplasmic reticulum Ca^2+^-ATPase
TEM: Tris-EDTA medium

## Supporting information

http://dx.doi.org/10.1111/bph.12685

**Table S1** Xestospongins ineffectively inhibit IP_3_-evoked Ca^2+^ release.
