# Supplementary material for: Interactions of antagonists with subtypes of inositol 1,4,5-trisphosphate (IP3) receptor
Source: Br J Pharmacol. 2014 Jun 10;171(13):3298–312. doi: 10.1111/bph.12685 (PMC4080982; doi:10.1111/bph.12685)
Supplement: Supplementary file 1 — Table S1 Xestospongins ineffectively inhibit IP3-evoked Ca2+ release. [file bph0171-3298-SD1.docx]

**Supporting Information**

**Table S1** Xestospongins ineffectively inhibit IP_3_-evoked Ca^2+^ release

|  | Incubation | IP_3_R1 | | IP_3_R2 | | IP_3_R3 | |
| --- | --- | --- | --- | --- | --- | --- | --- |
|  |  | ΔpEC_50_  (/M) | ΔMax  (%) | ΔpEC_50_  (/M) | ΔMax  (%) | ΔpEC_50_  (/M) | ΔMax  (%) |
| XeC^a^ | 5 µM,  5 min | 0.09 ± 0.07 | 2 ± 1 | 0.30 ± 0.25 | 5 ± 6 | -0.04 ± 0.11 | 15 ± 7 |
| XeC^b^ | 5 µM,  5 min | -0.12 ± 0.12 | 2 ± 2 | -0.02 ± 0.07 | 0 ± 4 | 0.02 ± 0.09 | 3 ± 14 |
| XeC^b^ | 20 µM,  7 min | 0.08 ± 0.07 | 9 ± 3^*^ | -0.07 ± 0.07 | 2 ± 2 | 0.15 ± 0.02^*^ | 1 ± 3 |
| XeC^b^ | 20 µM,  12 min | 0.33 ± 0.18 | 4 ± 3 | -0.04 ± 0.04 | 1 ± 2 | 0.10 ± 0.05 | 2 ± 2 |
| XeC | 20 µM,  pooled | 0.21 ± 0.10^*^ | 6 ± 2^*^ | -0.06 ± 0.04 | 1 ± 1 | 0.12 ± 0.03^*^ | 1 ± 2 |
| XeD^a^ | 10 µM,  5 min | 0.28 ± 0.13 | 17 ± 6^*^ | 0.25 ± 0.22 | 2 ± 4 | 0.03 ± 0.04 | 12 ± 1^*^ |
| XeD^b^ | 10 µM,  5 min | -0.02 ± 0.10 | 15 ± 4^*^ | -0.07 ± 0.07 | 2 ± 3 | 0.16 ± 0.26 | 8 ± 8 |
| XeD^b^ | 20 µM,  7 min | 0.37 ± 0.11^*^ | 18 ± 5^*^ | -0.06 ± 0.04 | 5 ± 2 | 0.39 ± 0.10^*^ | 4 ± 2 |
| XeD^b^ | 20 µM,  12 min | 0.14 ± 0.12 | 18 ± 1^*^ | -0.23 ± 0.06 | 11 ± 5 | 0.03 ± 0.08 | -1 ± 0 |
| XeD | 20 µM,  pooled | 0.26 ± 0.09^*^ | 18 ± 2^*^ | -0.15 ± 0.05 | 8 ± 3^*^ | 0.21 ± 0.10^*^ | 2 ± 2 |

Permeabilized DT40-IP_3_R1-3 cells were incubated with Xestospongin C or D (XeC or XeD) at the concentrations shown and for the periods indicated before addition of IP_3_. The two sources of Xestospongins are identified: ^a^Calbiochem and ^b^purified as described (Gafni *et al.*, 1997). The pEC_50_ values for IP_3_ and the maximal Ca^2+^ release (Max) are each expressed relative to the response evoked in the absence of Xestospongin (Δ = Control – Response with Xestospongin). A positive value for Δ demonstrates inhibition of IP_3_-evoked Ca^2+^ release by Xestospongin. The pooled analysis combines results with 20 µM of either antagonist from 7- and 12-min incubations. Results are means ± SEM from 3 (6 for pooled analyses) experiments. ^*^Denotes a value significantly greater than 0 (*p* < 0.025, one-tailed test). The pooled analyses are reproduced in Table 3.
